# Secondary myeloid neoplasms after CD19 CAR T therapy in patients with refractory/relapsed B-cell lymphoma: Case series and review of literature

**DOI:** 10.3389/fimmu.2022.1063986

**Published:** 2023-01-13

**Authors:** Aiqi Zhao, Mingzhe Zhao, Wenbin Qian, Aibin Liang, Ping Li, Hui Liu

**Affiliations:** ^1^ Department of Hematology, The Second Affiliated Hospital, School of Medicine, Zhejiang University, Hangzhou, Zhejiang, China; ^2^ Department of Hematology, Jinhua Municipal Central Hospital, Jinhua, Zhejiang, China; ^3^ Department of Hematology, Tongji Hospital of Tongji University, Shanghai, China

**Keywords:** chimeric antigen receptor T cells (CAR T), cellular immunotherapy, late effects, refractory/relapsed B-cell lymphoma, second cancer, therapy-related myelodysplastic syndrome (t-MDS), therapy-related acute myeloid leukemia (t-AML)

## Abstract

**Background:**

Several chimeric antigen receptor T cells (CAR T) targeting CD19 have induced profound and prolonged remission for refractory/relapsed (R/R) B-cell lymphoma. The risk of secondary malignancies, especially myeloid neoplasms, is of particular concern in the CAR T community, which still remains unclear.

**Methods:**

Four patients with R/R B-cell lymphoma after CD19 CAR T therapy diagnosed with secondary myeloid neoplasms (SMN) from 2 hospitals in eastern China were presented, including 3 with myelodysplastic syndrome (MDS) and 1 with acute myeloid leukemia (AML). Using single-cell RNA sequencing (scRNA-seq), we compared the cellular components of bone marrow (BM) samples obtained from one of these MDS patients and a health donor. We also provided a review of recently published literature concerning SMN risk of CAR T therapy.

**Results:**

Relevant demographic, clinical, laboratory, therapeutic and outcome data were collected and presented by chart review. In our case series, the male-female ratio was 3.0 and the median age at MDS onset was 61.25 years old (range, 50-78). Median number of previous systemic therapies was 4.5 (range, 4-5), including autologous hematopoietic stem cell transplantation (auto-HSCT) in one patient. BM assessments prior to CAR T therapy confirmed normal hematopoiesis without myeloid neoplasms. Moreover, for 3 patients with SMN in our series, cytogenetic analysis predicted a relatively adverse outcome. In our experience and in the literature, treatment choices for the patients with SMN included allogeneic hematopoietic stem cell transplantation (allo-HSCT), hypomethylating agent (HMA), period filgrastim, transfusions and other supportive care. Finally, treatment responses of lymphoma, together with SMN, directly correlated with the overall survival of this community. Of note, it appeared that pathogenesis of MDS wasn’t associated with the CAR T toxicities, since all 4 patients experienced a pretty mild CRS of grade 1-2. Additionally, scRNA-seq analysis described the transcriptional alteration of CD34+ cells, identified 13 T/NK clusters, and also indicated increased cytotoxic T cells in MDS BM.

**Conclusion:**

Our study illustrated the onset and progression of SMN after CD19 CAR T therapy in patients with R/R B-cell lymphoma, which provides useful information of this uncommon later event.

## 1 Introduction

CD19-targeted **c**himeric antigen receptor T cells (CAR T) therapy has shown prominent efficacy in relapsed/refractory (R/R) B cell malignancies with rapid response and long term remission (1). Short-term complications such as cytokine release syndrome (CRS) or immune effector cell-associated neurotoxicity syndrome (ICANS), have been well reported with standard management protocols (2). However, the potential late adverse effects, especially for events that presented and/or persisted beyond 100 days after CAR T cells infusion, are still not well recognized and require ongoing surveillance (3).

The most common late adverse events associated with CAR T therapy consist of prolonged cytopenia, hypogammaglobulinemia and infections (2). In accordance, it has also raised the concern whether this novel therapy results in increased risk of secondary malignancies, which has not been well-described. So far, two different patterns of secondary tumor have been described. The first pattern includes non-melanoma skin cancer, melanoma, bladder cancer, glioblastoma multiforme, and multiple myeloma. The second pattern reported is therapy-related myeloid neoplasms (t-MN), primarily therapy-related myelodysplastic syndrome (t-MDS). Antecedent cytotoxic therapies, including chemotherapy and/or radiation, appear to be the critical factors for the development of t-MN, however, it still remains unclear whether CAR T itself or immunosuppressive microenvironment participates in the malignant clonal evolution.

In this retrospective study we presented four patients with R/R B-cell lymphoma who received CD19 CAR T therapy and developed t-MN thereafter, three cases with MDS and one with overt acute myeloid leukemia (AML). Moreover, scRNA-seq analysis showed the distinct cell populations and transcriptional alteration of the CD34+ hematopoietic cells in tMDS bone marrow (BM). Furthermore, we also reviewed the most recent literature to enhance our understanding of this later complication of CAR T therapy.

## 2 Patients and methods

### 2.1 Patients and clinical evaluation

We reported four patients with R/R B cell malignancies who developed SMN after CD19 CAR T therapy. Data of new cases including demographic data, medical history and physical examination, were collected by medical records from the Second Affiliated Hospital of Zhejiang University in Hangzhou, China and Tongji Hospital of Tongji University in Shanghai, China from February 2019 to July 2022. A total of 126 patients received CAR T therapy and 4 patients were diagnosed as SMN. Diagnosis of MDS was based on the minimal diagnostic criteria published by the international MDS working group (4). Likewise, diagnosis of AML was also based on the 5th edition of the WHO classification of AML (5). This study was conducted according the Declaration of Helsinki and approved by the local Ethical Committee. All patients signed informed consent prior to study inclusion.

### 2.2 Sample collection, scRNA library preparation and sequencing

BM cells were obtained from one of our t-MDS cases (patient 1) and an age-matched health donor (HD). All participants provided written informed consent. Biospecimen collection protocols complied with local guidelines and were approved by the ethics committee. scRNA-seq analysis was performed using the 10 x Genomics System and the Chromium Single Cell 3′ Reagent Kit V2 according to the manufacturer’s protocol (6). scRNA-seq libraries were sequenced with a 150-bp paired end reads format on an Illumina NovaSeq 6000 system.

### 2.3 scRNA-seq data analysis

Alignment, barcode assignment, and unique molecular identifier counting were performed using the Cell Ranger pipeline (http://software.10xgenomics.com/singlecell/overview/welcome) (7). Single-cell datasets of tMDS patients and HD were integrated using “merge” function in version 3.2.2 of Seurat R package (8). We filtered cells that have unique feature counts over 3000, less than 200, and ≥ 10% mitochondrial counts. The merged dataset was normalized using Seurat “NormalizedData” function with a global-scaling normalization method “LogNormalize”, and multiplied this by a scale factor (10,000 by default). And then scaled by performing Seurat “ScaleData” function with regression of the variation of “nCount_RNA” and “percent.mt”. Performing Seurat “JackStrawPlot” function and “ElbowPlot” function helped to select suitable dimensionality. Dimension reduction analysis was performed by Seurat “RunPCA” function, and non-linear dimensional reduction was performed by Seurat “RunUMAP” function. Gene Sets Enrichment Analysis (GSEA; http://software.broadinstitute.org/gsea) was used to interpret gene set enrichment and pathways of defined differentially expressed genes.

### 2.4 Search strategy for reported SMN patients after CAR T therapy

We conducted a review of literature about myeloid neoplasms following CAR T therapy by searching for indexed articles and published abstract until July 2022 in MEDLINE *via* PubMed and the National Library of Medicine using the following keywords: “CAR T cell therapy” OR its synonyms (“chimeric antigen receptor T cell therapy”), in combination with “myelodysplastic syndromes” or other terms for this unusual complication (including “myeloid neoplasms” OR “second neoplasms” OR “subsequent malignancies” OR “hematologic toxicity” OR “late effects” OR “late cytopenia” OR “prolonged cytopenia” OR “long-term complications”). We selected only studies including patients with a definite diagnosis of t-MN after receiving CAR T therapy.

## 3 Results

### 3.1 New case presentations

#### 3.1.1 Patient 1

**Figure 1:**
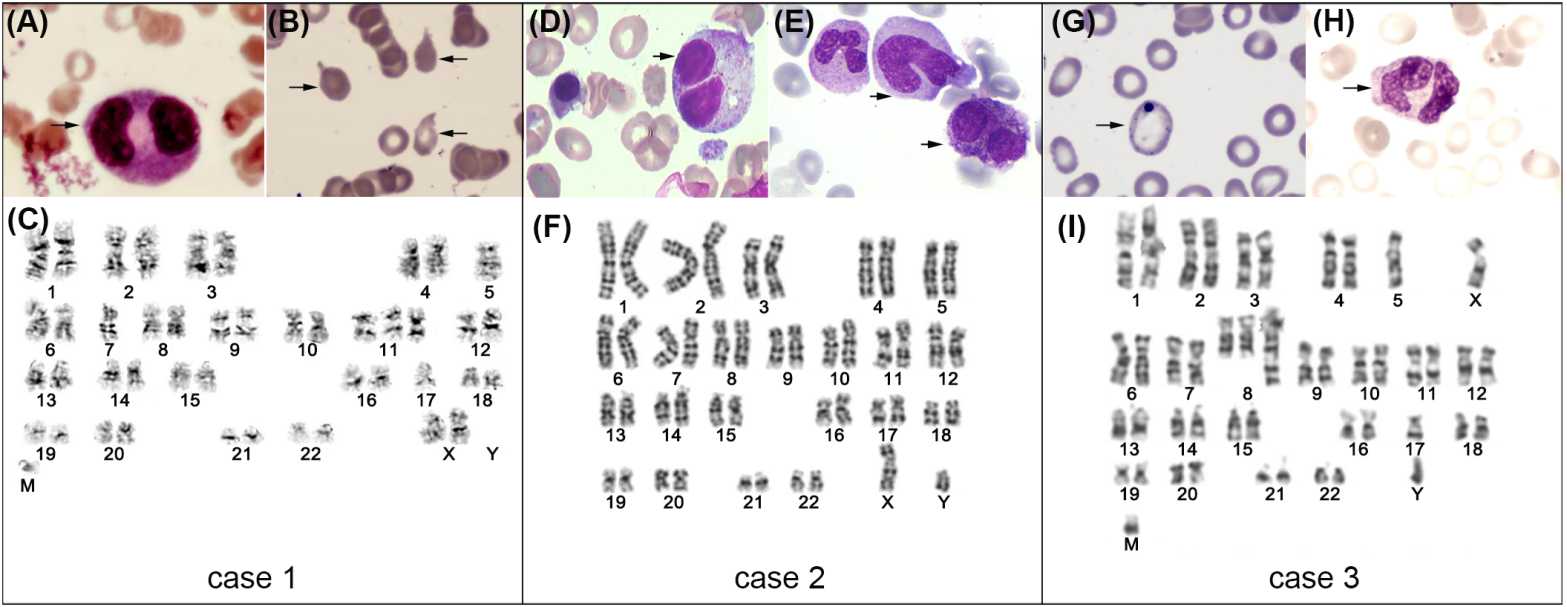
BMA of case 1~3 at diagnosis of MDS (representative images of BM cytomorphology and karyotype analysis). For case 1 **(A–C)**: binuclear myelocyte **(A)**, teardrops **(B)** and karyotype analysis depicting 45, XX, -5, -7, +add (11) (p15), -17, +mar **(C)**. For case 2 (D~F): binuclear myelocyte **(D)**, giant neutrophils and hyposegmented neutrophils **(E)**, and karyotype analysis depicting 46, XY **(F)**. For case 3 **(G–I)**: basophilic stippling of red cells **(G)**, binuclear myelocyte **(H)**, and karyotype analysis depicting 42~46, XY, del (3) (p24), -5, +add (8) (q24.1), -17, add (18) (q23), +mar, inc[cp9]/46, XY [11].

A 53-year-old Chinese female patient was diagnosed with follicular lymphoma (grade 1, stage II) in January 2007. BM assessment (BMA) such as biopsy and aspirate analysis (including flow cytometry (FCM) and cytogenetic analyses) was normal at diagnosis. After first-line therapy of R-CHOP, she achieved CR. Unfortunately, her disease relapsed and transformed to DLBCL in March 2014, according to the immunohistochemistry analysis (IHC) of cervical lymph node biopsy showing CD19 (+), CD20 (+), CD3 (-), CD5 (-), CD10 (+), CD45 (+), BCL2 (+), BCL6 (+), MUM1 (+), MYC (+) and Ki-67 (+, 80%). Over the following five years, she had received multiple lines of regimens, however, disease progressed again in May 2019. Immunophenotyping using IHC of lymphoma tissue was strongly positive for CD19. Eventually she was enrolled in the clinical trial of the fourth generation CD19 CAR T expressing IL-7 and CCL19 (NCT04833504). After lymphodepletion, she received cell infusion in July 2019. Within the first two weeks, grade 2 CRS was developed and quickly resolved with methylprednisolone and tocilizumab. Thereafter routine follow-up evaluation was performed every 3 months and lymphoma remained CR for more than 2 years. She didn’t exhibit prolonged cytopenia during the initial 2 years. Whereas, she developed persistent and exacerbating pancytopenia thereafter. Eventually, BMA supported the diagnosis of MDS with multilineage dysplasia (MDS-MLD) in January 2022. As shown in **Figures 1A–C**, the morphological assessment exhibited decreased cellularity, dysplasia of erythroid and myeloid cells (mild anisocytosis, teardrop cells, and binuclear myelocytes) and increased lymphoid cells; FCM determined the marrow blast percentage of 0.3% and growing lymphoid percentage of 62%; cytogenetics ascertained the abnormal karyotype of 45, XX, -5, -7, +add (9) (p15), -17, +mar [15]/46, XX [5]; MDS-associated genes revealed the TP53 mutation (c.711G>A, variant allele frequency (VAF) of 9.85%) and GATA2 mutation (c.848G>A, VAF of 45.58%). According to the Revised International Prognostic Scoring System (IPSS-R), she was classified in the very high-risk MDS category. Based on the patient’s performance status and her own preference, she refused the therapeutic option of transplant. Systemic management strategies were implemented including the hypomethylating agent (HMA) azacytidine (AZA, 75mg/m^2^/day for 7 days every 28 days) and immunomodulatory drugs thalidomide (50mg/day), together with supportive care including recombinant human erythropoietin (EPO), filgrastim and transfusions. After three cycles of AZA, transient hematologic improvement had ever been observed based on IWG criteria (10). Unfortunately, after having been diagnosed as MDS for 8 months, she died from aggravating gastrointestinal hemorrhage and pulmonary infection due to worsening pancytopenia (details shown in **Table 1**).

**Table 1:** New patients with SMN after CAR T therapy.

Patient NO.	1	2	3	4
Lymphoma				
Age (year)	53	64	78	50
Gender	F	M	M	M
ECOG PS	2	3	3	2
Subtype	TFL	DLBCL/non-GCB	DLBCL/GCB	DLBCL/non-GCB
Stage	IV	IV	IV	IV
B symptom	A	B	A	B
Target lesion	Lungs, pleura, breast, lymph nodes (9 regions)	Lungs, lymph nodes (6 regions)	Parotid gland, lymph nodes (6 regions)	Adrenal glands, bone, lymph nodes (10 regions)
BMA at diagnosis	Normal	Normal	Normal	Normal
Prior therapies				
System regimens	R-CHOP, R-ICE, EPOCH, HyperCVAD	R-CHOP, R-GDP, R-MINE, R-DHAP, lenalidomide	R-CHOP, R-GDP, lenalidomide, zanubrutinib	R-CHOP, R-ICE, lenalidomide, ibrutinib
Prior RT	No	No	Yes	No
Prior auto-HSCT	No	No	No	Yes
CAR T therapy				
Cytopenia before CAR T	No	No	thrombocytopenia, anemia	No
BMA before CAR T	NA	NA	Erythroblast (1%) morphology and FCM: normal	NA
Fludarabine dose (mg/m^2^ × 3 days)	30	*1st infusion:* 30 *2nd infusion:* 30	25	30
CTX dose (mg/m^2^ × 3 days)	500	*1st infusion:* 500 *2nd infusion:* 500	300	300
CAR T products	CD19 CAR T expressing IL-7 and CCL19	*1st infusion:* CD19 CAR T (4-1BB based) *2nd infusion:* CD19 PD-1/CD28-CAR T	relma-cel (commercial use)	CD20/CD19 bispecific CAR T
Dose (10^6^/kg)	1	*1st infusion:* 2 *2nd infusion:* 2	5	5
CRS max grade	2	*1st infusion:* 1 *2nd infusion:* 2	2	2
CRS duration (day)	11	*1st infusion:* 5 *2nd infusion:* 9	10	5
ICANS	No	No	No	No
Cytopenia after CAR T	No	1st infusion: No 2nd infusion: Yes	Yes	Yes
Best response	CR	CR	PR	PR
Final response	CR	CR	PR	PR
SMN				
Subtype	MDS-MLD	MDS-MLD	MDS-MLD	AML-M4
Onset after first CAR-T (month)	30	78	3	3
Risk (IPSS-R or ENL)	very-high risk	intermediate-risk	very-high risk	poor-risk
Cytogenetics	-5, -7, +add (11) (p15), -17, +mar	46, XY	del (3) (p24), -5, +add (8) (q24.1), -17, add (18) (q23), +mar, inc[cp9]	t (4;11) (p16; q23), del (5) (q13q22)
Mutations	TP53, GATA2	TET2	TP53, DNMT3A	TET2, WT1, CEBPA
Treatment	AZA, thalidomide, filgastrim, EPO and transfusions	filgastrim, EPO and transfusions	filgastrim, EPO and transfusions	standard-dose cytarabine with idarubicin, AZA, venetoclax, low-dose cytarabine-containing regimens
Best response	Hematologic improvement	No response	No response	CRi
Final response	Progression	Failure	Failure	Relapse
Outcome				
Follow-up since SMN (month)	8	5	3	20
Lymphoma	CR	CR	PR	PR
SMN	death related to MDS	death related to MDS	death related to MDS	Survival, relapse

auto-HSCT, autologous hematopoietic stem cell transplantation; AZA, azacytidine; BMA, bone marrow assessment; CHOP, cyclophosphamide, adriamycin, vincristine, prednisone; CRS, cytokine release syndrome; CR, complete remission; CRi, complete remission with incomplete hematologic recovery; CTX, cyclophosphamide; DHAP, dexamethasone, high dose cytarabine, cisplatin; DLBCL, diffuse large B cell lymphoma; ECOG PS, Eastern Cooperative Oncology Group performance status; ENL, the European LeukemiaNet recommendations for risk stratification by genetics; EPO, recombinant human erythropoietin; EPOCH, etoposide, prednisone, vincristine, cyclophosphamide, doxorubicin; FCM: flow cytometry; GCB, germinal center B cell; GDP, gemcitabine, dexamethasone, cisplatin; HyperCVAD, cyclophosphamide, vincristine, doxorubicin, dexamethasone; ICANS, immune effector cell-associated neurotoxicity syndrome; ICE, ifosfamide, carboplatin, etoposide; IPSS-R, the Revised International Prognostic Scoring System; MINE, mesna, ifosfamide, mitoxantrone, etoposide; NA, not available; PR, partial remission; PS, performance status; RT, radiation therapy; SMN, secondary myeloid neoplasms; TFL, transformed follicular lymphoma; R, rituximab; relma-cel, relmacabtagene autoleucel.

#### 3.1.2 Patient 2

A 64-year-old Chinese male was diagnosed as DLBCL (non-GCB, stage IV) in June 2015, based on the IHC analysis of cervical lymph node biopsy showing CD19 (+), CD20 (+), CD3 (-), CD5 (-), CD10 (-), CD45 (+), BCL2 (+), BCL6 (-), MUM1 (+), MYC (-) and Ki-67 (+, 80%). BMA showed normal hematopoiesis at diagnosis. After receiving multiple lines of chemotherapies, he didn’t obtain any response and then was enrolled in the clinical trial of CD19 CAR T therapy with 4-1BB for co-stimulation (NCT 02644655). After lymphodepletion, he received cell infusion in January 2016. Grade 1 CRS was developed and resolved within the first week by use of ibuprofen and intravenous (IV) fluids maintenance. Thereafter lymphoma had remained CR for more than 3 years by August 2019, when his cervical lymph node enlarged again and was confirmed as the recurrence through open biopsy and PET/CT scan. Then he received the second infusion of CD19 CAR T cells that express a PD-1/CD28 chimeric switch-receptor (CD19-PD-1/CD28-CAR T) as a salvage treatment (NCT04850560) in July 2019. Within the initial two weeks, grade 2 CRS developed and was controlled by methylprednisolone and tocilizumab. Thereafter his lymphoma achieved sustained CR for more than 2 years. After second infusion, grade 1 neutropenia and grade 1 thrombocytopenia had been persisted according to National Cancer Institute Common Terminology Criteria for Adverse Events (NCI CTCAE) version 5.0, which was attributed to the hematopoietic toxicity of CAR T therapy. However, since January 2022, his cytopenia was aggravating and BMA supported the diagnosis of MDS-MLD in May 2022, based on the minimal diagnosis criteria (4). Morphologic assessment showed increased marrow cellularity and dysmorphic cells, especially very large and small erythroblasts, hypolobulated neutrophil and asymmetric binucleation (**Figures 1D, E**); FCM showed 1.49% blasts and 39.9% lymphoid cells in BM; the karyotype analysis showed 46, XY (**Figure 1F**); MDS-associated genes panel revealed the TET2 mutation (c.3986T>C, VAF of 35.4%; c.4100C>G, VAF of 37.3%; c.4250T>G, VAF of 2%). According to the IPSS-R, he was classified into the intermediate-risk MDS category. Patients who fall under the IPSS-R intermediate category can be managed as either lower-risk patients or higher-risk patients. Since he refused the use of any cytotoxic therapies, he was administered with periodic filgrastim and EPO, and consistently depended on transfusion products. After having been diagnosed as MDS for 5 months, he died from respiratory failure due to aggravating pulmonary infection (details shown in **Table 1**).

#### 3.1.3 Patient 3

A 78-year-old male patient was diagnosed with DLBCL (GCB, stage IV) in July 2021 according to the IHC analysis of parotid gland biopsy showing CD19 (+), CD20 (+), CD3 (-), CD5 (-), CD10 (-), CD45 (+), BCL2 (-), BCL6 (+), MUM1 (-), MYC (+) and Ki-67 (+, 90%). BMA showed normal hematopoiesis at diagnosis. After receiving multiple lines of therapies, his disease remained progression and he decided to receive commercial relmacabtagene autoleucel (relma-cel) infusion. Considering he exhibited grade 3 thrombocytopenia and grade 2 anemia, BMA was processed before infusion, which indicated significantly reduced erythroblast (1%), normal morphology and normal immunophenotyping. Regrettably, the cytogenetic data was scarce. After lymphodepletion, he received relma-cel infusion in April 2022. Within the initial two weeks, he experienced grade 2 CRS which was ameliorated by methylprednisolone and tocilizumab. Whereas, he developed persistent pancytopenia during the following 3 months, showing ≥grade 3 anemia, ≥grade 3 thrombocytopenia and ≥grade 3 neutropenia. BMA was repeated twice and supported the diagnosis of MDS-MLD in June 2022. As shown in **Figures 1G–I**, the morphological assessment exhibited increased cellularity and dysplasia of erythroid and myeloid cells (hypogranular neutrophils, abnormal red blood cells (RBC) of mild anisocytosis and basophilic stippling); FCM showed 11.5% lymphoid cells in the BM; cytogenetics ascertained the abnormal karyotype of 42~46, XY, del (3) (p24), -5, +add (8) (q24.1), -17, add (18) (q23), +mar, inc[cp9]/46, XY [11]; MDS-associated genes revealed the TP53 mutation (c.818G>A, VAF of 45.3%, c.659A>G, VAF of 17%, c.636del, VAF of 11.7%) and DNMT3A mutation (c.939G>A, VAF of 13.3%). According to IPSS-R, he was classified in the very high-risk MDS category. On the evidence of MDS diagnosis, systemic management strategy of HMA (decitabine, 20mg//m^2^/day for 5 days every 28 days) was initiated soon, together with supportive care. Although two cycles of decitabine had been used, no evident hematologic improvement was observed. Besides, primary lymphoma achieved PR. After having been diagnosed as MDS for 3 months, he died from septic shock due to exacerbating pulmonary infection (details shown in **Table 1**).

#### 3.1.4 Patient 4

A 50-year-old male patient was diagnosed with DLBCL (non-GCB, stage IV) in December 2018 according to the immunohistochemistry analysis of parotid gland biopsy showing CD19 (+), CD20 (+), CD3 (-), CD5 (-), CD10 (-), CD45 (+), BCL2 (-), BCL6 (+), MUM1 (+), MYC (+) and Ki-67 (+, 90%) (details shown in **Table 1**). BMA showed normal hematopoiesis at diagnosis. After first-line therapy of R-CHOP and autologous hematopoietic stem cell transplantation (auto-HSCT), he achieved CR in September 2019. However, his disease relapsed in April 2020. Though implementation of multiple lines of chemotherapeutics, his disease still progressed. Eventually he was enrolled in the clinical trial of second generation anti-CD20/CD19 bi-specific CAR T therapy (NCT04317885). After lymphodepletion, he received cell infusion in November 2020. Within the first week, he experienced grade 2 CRS which was ameliorated by methylprednisolone and tocilizumab. However, he was diagnosed with AML-M4, poor-risk in February 2021 per European LeukemiaNet (ELN) recommendations (11). Based on the BMA results, morphological assessment showed approximately 33 percent of marrow nucleated cells are myeloblastic and monoblastic leukemic cells. They are positive for peroxidase, Sudan, chloroacetate esterase, and non-specific esterase, partially inhibited by NaF. FCM showed MPO++, CD33+, CD13+, CD117+, CD64+, CD34+, CD38+, HLA-DR+, CD123+, CD56++, CD7+. Cytogenetics ascertained the abnormal karyotype of 46, XY, t (4;11) (p16; q23), del (5) (q13q22) [18]/46, XY [2]. Molecular analysis revealed the CEBPA mutation (c.339_343del, VAF of 50.1%), TET2 mutation (c.3688A>T, VAF of 36.2%) and WT1 mutation (c.1156_1159dup, VAF of 28%). Thereafter, he received induction therapy of standard-dose cytarabine and idarubicin but resulting in subarachnoid hemorrhage. The follow-up BMA indicated CR with incomplete hematologic recovery (CRi), with leukemic blasts of 4.5% in the marrow. Over the following period of one year and a half, he had been administered with lower-intensity approaches comprising AZA, venetoclax, and low-dose cytarabine-containing regimens. However, he never reached CR again. Currently, he has been followed-up for 20 months since his diagnosis of AML and he still remained PR for primary lymphoma (details shown in **Table 1**).

### 3.2 scRNA-seq-based investigation of a tMDS BM sample after CAR T therapy

#### 3.2.1 Identification of cell populations in tMDS and normal BM samples

**Figure 2:**
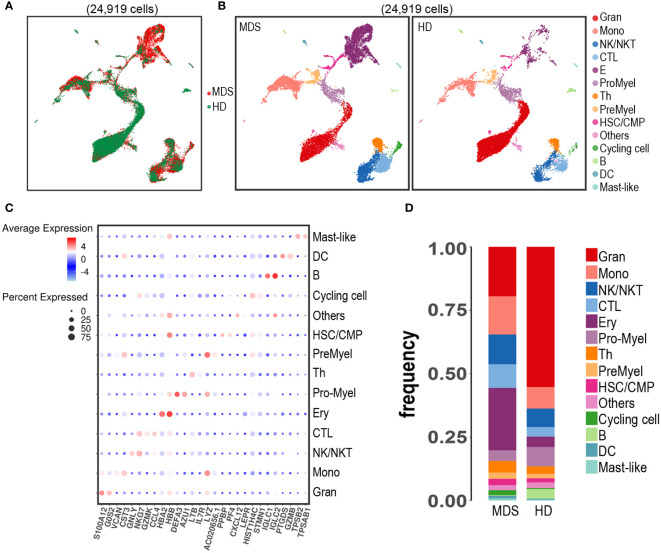
Identification of cell populations in BM samples from patient 1 developing tMDS after CAR T therapy and an age-matched HD using scRNA-seq. UMAP projection of scRNA-seq data for 24,919 hematopoietic cells from patient 1 and HD based on 10 principal component analysis consolidations and the 1,000 most variable genes in the data set. **(A)** t-SNE plot of all the 24,919 single cells from tMDS patient and HD. Each dot represents a single cell; the dot colors indicate source of donors. **(B)** t-SNE plot of all the 24,919 single cells colored by predicted hematopoietic populations in tMDS patient and HD. Cells were annotated based on known linease-specific marker genes. **(C)** Dot plot of differentially key cell-type marker genes. **(D)** Stacked barplots show the frequencies of 14 cell types in bone marrow from tMDS patient and HD, colored based on cell type.

In total, after quality control, 13,939 and 10,980 single cell libraries from a tMDS patient and HD, respectively, were retained for further analysis, as shown in **Figure 2A**. The scRNA-seq data from BM cells was performed uniform manifold approximation and projection (UMAP) analysis. These 24,919 BM-derived cells were segregated into 14 populations (**Figure 2B**) based on expression of established marker genes (**Figure 2C**). All 14 cell types were identified in both HD and tMDS patient, suggesting that much of the transcriptional hierarchy of normal BM lineage populations was still reflected in this tMDS patient. The frequencies of different cell types in tMDS and normal BM were also shown in **Figure 2D**. Despite interpatient heterogeneity, patient 1 exhibited higher proportions of multiple immune cells in BM, including cytotoxic T cells (CTL), T helper cells (Th), NK/NKT cell, monocyte and DC, and lower frequencies of B cell and granulocyte subsets.

#### 3.2.2 Transcriptional alteration of the hematopoietic stem cell/common myeloid progenitor (HSC/CMP) population in the tMDS patient

**Figure 3:**
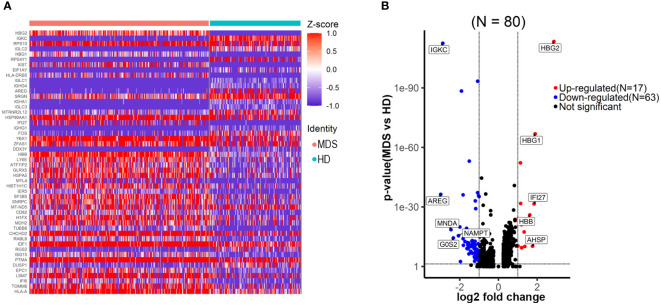
Transcriptional changes of the HSPC/CMP population in the tMDS patient identified with scRNA-seq. **(A)** Heatmap of the top 50 significantly differentially expressed genes between t-MDS versus HD HSPC/CMP populations, highlighting the distribution of transcript expression within the clusters. **(B)** Volcano plot revealing significantly differentially expressed genes between t-MDS versus HD HSPC/CMP populations (FDR ≤ 0.05 and log2 fold change≥1.0, ≤-1.0).

Differential gene expression analysis was performed within the HSC/CMP population based on CD34 expression and UMAP signatures. We identified 80 genes that were significantly differentially expressed (log2 fold change ≥1.0, ≤-1.0, false discovery rate [FDR] ≤ 0.05) (**Figures 3A, B**; **Supplementary Table 1**), with genes upregulated (n=17) and downregulated (n=63). We noted upregulation of a set of previously reported up-regulated genes in MN, including HBG2 (9), HBG1 (9), IFI27 (12), AHSP (13–15), PRDX2 (16) and BLVRB (17). Moreover, a separate GSEA analysis revealed significant (FDR ≤ 0.05) positive enrichment of gene sets involving in oxidative stress, metabolism-related, RNA processing and erythropoiesis; negative enrichment of gene sets involving in immune-related pathways (**Table 2**), consistent with previous reports (9, 18).

**Table 2:** GSEA enriched pathways in HSC/CMP population.

Upregulated pathways	SIZE	ES	NES	p-value
**Oxidative stress**				
hydrogen peroxide catabolic process	10	0.792	2.608	0.000
hydrogen peroxide metabolic process	16	0.613	2.502	0.000
**Metabolism-related**				
heme metabolic process	13	0.649	2.365	0.000
tetrapyrrole metabolic process	14	0.603	2.307	0.000
mitochondrial gene expression	19	0.531	2.260	0.000
**RNA processing**				
spliceosome snRNP assembly	10	0.639	2.103	0.001
RNA localization to Cajal body	10	0.592	1.947	0.006
**Erythropoiesis**				
heme biosynthetic process	12	0.586	2.073	0.003

Downregulated pathways	SIZE	ES	NES	p-value
**Immune**				
Cytokine signaling	11	-0.789	-2.357	0.000
Defense response to fungus	13	-0.715	-2.265	0.000
regulation of lymphocyte proliferation	14	-0.686	-2.218	0.000
neuroinflammatory response	15	-0.669	-2.237	0.000
monocyte chemotaxis	10	-0.650	-1.882	0.004
humoral immune response	15	-0.614	-2.054	0.000
B cell receptor signaling pathway	20	-0.613	-2.242	0.000

#### 3.2.3 T/NK cell subpopulations analysis

**Figure 4:**
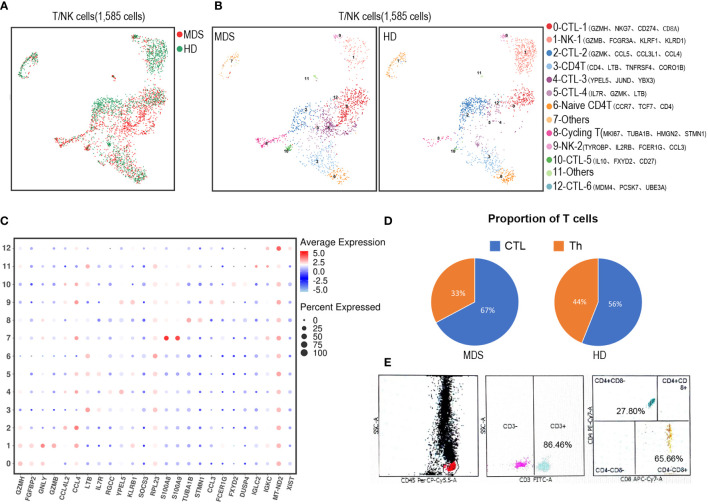
Dissection and clustering of T/NK subpopulations in the tMDS patient and HD. **(A)** t-SNE plot shows all identified T/NK subpopulations in tMDS and normal BM samples. The dot colored by source of donors. **(B)** UMAP plot of T/NK subpopulations from Figure 2B-represented CTL, Th and NK/NKT subsets. These T/NK cells can be divided into 13 subsets. **(C)** Dot plot of differently key cell-type marker genes. **(D)** Pie charts show relative ratio of cells annotated as CTL or Th cells in BM samples by scRNA-seq. **(E)** FCM analysis for T cells (CD3, CD4 and CD8) in the tMDS BM sample.

To uncover the spectrum of T/NK heterogeneity and states, we utilized UMAP to re-cluster 1,585 T/NK-like cells (**Figure 4A**) with more details. Thus, the T/NK lineage was divided into 13 clusters (**Figure 4B, C**). CD4+ T cells (Th) were dissected into 2 subsets (cluster 3 and 6); CD8+ T cells (CTL) were dissected into 6 subsets (cluster 0, 2, 4, 5, 10 and 12); NK cells were dissected into 2 subsets (cluster 1 and 9). Among the above, CD4+ naïve T cells (Cluster 6) were identified by the high expression of naïve/TCM-state-related genes (CCR7 and TCF7). Cycling T cells (cluster 8) expressed canonical proliferation markers MKI67, TUBA1B and STMN1. However, the limited cell numbers precluded further analysis, for example, identification of the suppressive T regulatory cell (Treg) subset, which was considered to be increased in the immunosuppressive BM environment of MDS. Besides, the tMDS aspirate tended to have proportionally more CTL and less Th than normal BM (**Figure 4D**). In accordance, the FCM analysis of this tMDS BM also showed an elevated CTL/T cell ratio of 65.66% (**Figure 4E**). Thus, scRNA-seq and FCM analysis revealed the consistent change in T cell composition in tMDS BM after CAR T therapy.

## 4 Literature review

**Table 3** summarized a literature review of SMN after CD19 CAR T therapy. Since January 2019, it was found that six clinical researches made references to the development of SMN during the long-term follow-up (19–24). Of note, all data of SMN was observational. The incidence of SMN after CD19 CAR T therapy was reported at varying rates ranging from 0.9% to 12.9% across studies, while the etiology of this observation might be problematic and multifactorial. In the largest series evaluating 1297 patients with R/R large B-cell lymphoma (LBCL) receiving commercial axicabtagene ciloleucel (axi-cel) in real-world practice from the Center for International Blood and Marrow Transplant Research (CIBMTR), 15 patients were diagnosed as MDS over the median follow-up of 12.9 months, with a incidence of 1.1% (24). While another study from MD Anderson Cancer Center reported the highest MDS incidence of 12.9% (4/31) from ZUMA-1 and ZUMA-9 (23), after a median of 13.5 months (range, 4-26 months). Of note, in this cohort, there was no statistical significantly difference in the MDS incidence between patients with or without prolonged cytopenia after cell infusion (3/15 [20%] vs. 1/16 [6%], p=0.33).

**Table 3:** SMN in a sampling of CD19 CAR T studies.

SMN N (%)	Sample Size	Lymphoid Malignancies	CAR T Used	Onset of MDS (month)	Associated Clinical Features	Treatment	Lymphoma Outcome	MDS Outcome	Reference
1 MDS (0.9%)	108	R/R DLBCL, PMBCL, TFL	Axi-cel (ZUMA-1)	18.9	NA	NA	NA	NA	*Locke et al. ^(19)^*
4 MDS (4.6%)	86	R/R B-ALL, NHL, CLL	CD19 CAR with 4-1BB co-stimulatory domain	6 (range, 4-17)	1) RAEB-2 2) NA 3) NA 4) NA	1) Ara-C 2) AZA 3) allo-HSCT 4) decitabine	1) dead 2) CR 3) NA, alive 4) CR	1) dead 2) NA, alive 3) NA, alive 4) NA, alive	*Cordeiro et al. ^(20)^*
2 MDS (9%)	22	R/R DLBCL	Axi-cel (commercial use)	NA	1) RAEB-2, 7q-, PPM1D mutation 2) MDS-MLD, low IPSS 0.5, 20q-	1) dead 2) filgastrim and supportive care	1) PD, dead 2) CR	1) dead 2) alive	*Nahas et al. ^(21)^*
2 MDS (1.4%)	137	R/R B-ALL	Tisa-gen (ELIANA and ENSIGN)	NA	NA	NA	NA	1) NA 2) resolved	*Levine et al. ^(22)^*
4 MDS (12.9%)	31	R/R DLBCL, PMBCL, TFL	Axi-cel (ZUMA-1 and ZUMA-9)	13.5 (range, 4-26)	NA	NA	NA	NA	*Strati et al. ^(23)^*
15 MDS (1.1%)	1297	R/R DLBCL, PMBCL and HGBL	Axi-cel (commercial use)	NA	NA	NA	NA	NA	*Jacobson et al. ^(24)^*

allo-HSCT, allogeneic hematopoietic stem cell transplantation; AML, acute myeloid leukemia; Axi-cel, axicabtagene ciloleucel; AZA, azacytidine; B-ALL, B cell acute lymphoblastic leukemia; CAR T, chimeric antigen receptor T cells; CLL, chronic lymphocytic leukemia; CR, complete remission; HGBL, high-grade B-cell lymphoma; MDS, myelodysplastic syndrome; NA, not available; NHL, Non-Hodgkin lymphoma; PD, disease progression; PMBCL, primary mediastinal B-cell lymphoma; R/R DLBCL, refractory/relapsed diffuse large B cell lymphoma; RRMM, relapsed or refractory multiple myeloma; SMN, secondary myeloid neoplasms; TFL, transformed follicular lymphoma; Tisa-gen, tisagenlecleucel.

Not surprisingly, the heavy load of pretreatment for primary malignancies prior to CAR T therapy is of particular concern in the SMN population, because of the increasing risk of mutagenesis which raise the possibility of secondary malignancies. Data from MD Anderson Cancer Center which reported the highest incidence of SMN, described the median number of previous systemic therapies in four MDS patients was 5 (range, 4-7), including auto-HSCT in one patient (23). Notably, no myeloid driver mutation was detected in this MDS cohort before lymphodepletion. Nahas and Cordeiro et al. also reported the same median number 5 (range, 3-7) (20, 21). Specifically, Nahas et al. described the cytopenia history before cell infusion in both MDS patients, while there was no evidence supporting existence of clonal hematopoiesis of indeterminate potential (CHIP) before CAR T therapy. In addition, Cordeiro et al. demonstrated that 2/4 MDS patients had cytogenetic abnormalities prior to CAR T therapy and 2/4 MDS patients had ever experienced auto-HSCT before.

As shown in **Table 1**, axicabtagene-ciloleucel (axi-cel) was used in four out of the six studies, including clinical trials and real-world practice, while tisagenlecleucel (tisa-gen) and locally produced second generation CD19 CAR T with 4-1BB co-stimulatory domain in the other two studies. Nahas et al. reported that CRS was mild in two MDS patients, with one grade 1 CRS and one grade 2 CRS (21). However, the max CRS grades were not described in other five studies.

The median time from the first CAR T cell infusion to diagnosis of SMN was 10.7 months (range, 4-26). Subsequent MDS had heterogeneous severity with some patients requiring periodic HMA administration and receiving allogeneic hematopoietic stem cell transplantation (allo-HSCT), while some experiencing only transient dysplasia and completely resolving within 3.5 months (22). Cordeiro et al. (20) reported the treatment strategies and outcomes in 4 MDS patients, which suggested that HMA and allo-HSCT still played an important role in treatment options for SMN, despite the small sample size. The first patient received periodic decitabine and had been alive for more than 10 months, with CR for lymphoma; the second patient received AZA, and had been alive for more than 40 months, also with CR for lymphoma; the third patient just finished allo-HSCT less than one month; the fourth patient with RAEB-2 was treated with high dose Ara-C, and died from uncontrolled MDS and active lymphoma. Similarly, in the SMN cohort reported by Nahas et al. (21), one patient was died from progressive DLBCL; the other was administered with periodic filgrastim for MDS, and remained CR for DLBCL. Collectively, it appeared to be evident that overall survival of SMN patients was closely associated with both the management of MDS and primary lymphoma.

## 5 Discussion

Currently, late adverse events of CAR T therapy are not fully understood, especially for second cancers (25). With a goal of increasing our understanding of one of the most common second cancers, we elaborated a case series of SMN after CAR T therapy in patients with R/R B-cell lymphoma, accompanied by a review of literature.

SMN following CAR T therapy should be classified as a sort of t-MN per 2022 WHO-classification, which refers to patients who develop MN following cytotoxic therapy and can be further divided into 2 subgroups: t-MDS and t-AML (26). Actually, in clinical practice, the diagnosis, prognosis staging, and therapy regimens of t-MDS/t-AML are made following the same criteria as for primitive MDS (p-MDS) or *de novo* AML, exclusive of a previous history of non-myeloid neoplasm (27). In comparison with p-MDS, t-MDS has always been recognized to induce poorer prognosis and most patients belong to high or very high-risk categories (28). Regrettably, since t-MDS patients have always been excluded from therapeutic clinical trials, there is little experience of application of novel agents in t-MDS and the only curative approach for t-MDS remains allo-HSCT. Similarly, only a few clinical studies focused on t-AML, primarily based on subset analysis of larger studies or extrapolation. For example, HMA (29, 30) and the combination of venetoclax have been suggested for management of t-MDS/t-AML. Given that most patients post CAR T therapy are not candidates for allo-HSCT due to age, fitness, comorbidities or infection, HMA is likely to constitute a potential treatment modality instead. Supportive of this, a retrospective study suggested the activity of HMA in t-MDS was roughly comparable to what was reported in p-MDS (overall response rate 38%, median overall survival 9.2 months) (31).

On the other hand, accumulating preclinical studies have confirmed epigenetic modulation could enhance CAR T cell persistence and function and trafficking with the immunosuppressive tumor microenvironment. Various modalities combining HMA with CAR T therapy have been evaluated and gained marked efficacy, irrespective of almost all data from phase I or II clinical trials (32). We confirmed that antitumor functions of CD123 CAR T cells were significantly enhanced by low-dose decitabine in AML patients (33). Additionally, Wang et al. (34) demonstrated that low-dose decitabine priming endowed CAR T cells with stronger anti-lymphoma, proliferation and cytokine-releasing capacities. Collectively, among t-MN patients after CAR T therapy, it seems as if that HMA constitutes a potential approach to ameliorating the poor prognosis of t-MN as well as to maintaining durable response of CAR T therapy for R/R B-cell lymphoma.

In our four new cases, the male-female ratio was 3.0 and the median age at MDS onset was 61.25 years old (range, 50-78). 3 suffered from R/R DLBCL and 1 suffered from TFL. The median number of previous systemic therapies was 4.5 (range, 4-5). As for literature review, the median number of previous therapies from three institutes was 5 (range, 3-7) (19) (20) (23). We further analyzed the prior cytotoxic therapies in our cases. Of note, all cases received the first-line chemotherapy of CHOP and preferred lymphodepletion of FC, which contained both alkylating agents (cyclophosphamide, fludarabine) and topoisomerase II inhibitors (anthracyclines); besides, one patient received radiotherapy; two patients received target agents, BTK inhibitors (BTKi) and lenalidomide; one patient received auto-HSCT prior to CAR T therapy. Our cases and literature review suggested the heavy load of pretreatment was an evident similarity among t-MN population, which was likely to correlate with the etiology of t-MN. The supportive evidence is as follows.

First and foremost, a panel of studies have demonstrated that different treatment modalities in NHL are associated with a higher risk of t-MN (35). For a start, it has been clearly demonstrated that conventional chemotherapy increases the risk, among which alkylating agents and topoisomerase II inhibitors in particular are known to be causative (36). Three large trials described the incidence of t-MN in NHL patients receiving conventional chemotherapy was in the range of 5% to 8% at 10 years (35). Subsequently, low-dose TBI was also demonstrated to be leukemogenic and the combination of low-dose TBI and alkylating agents had a synergistic effect, with the t-MN incidence of 12% for 41 patients at a median follow-up of 9.7 years reported by Travis et al. (37). Moreover, the contribution of auto-HSCT to t-MN incidence is less certain, given the heterogeneity in different studies. According to a clinical study enrolling 4998 transplanted patients, for NHL patients, the incidence of t-MN was 0.7% (0.4–1.2), 3.0% (CI 2.0–4.3) and 5.7% (CI 3.4–9.4) at 2, 4 and 10 years separately (38), which was similar to incidence in patients treated with conventional chemotherapy. Several studies confirmed a similar conclusion (26), which supported the hypothesis that much of the risk for t-MN was determined by pre-transplant therapies, rather than auto-HSCT. Lastly, though new agents have been rapidly altering the treatment paradigms, data for t-MN is relatively limited. Woyach et al. (39). reported the second cancer incidence in CLL patients receiving BTKi after a median follow-up of 44 months, 6/691 patients were diagnosed with t-MN and all cases had ever been treated with cytotoxic agents before. As for lenalidomide, since there is still limited experience in lymphoma patients, all t-MDS/t-AML cases were described in multiple myeloma patients (40). Collectively, despite the complexity and heterogeneity of pretreatment for R/R B cell lymphoma, it is reasonable to postulate that the heavy pretreatment is implicated in the pathogenesis of t-MN for patients receiving CAR T therapy.

Moreover, it is interesting to note that t-MN incidence after CAR T therapy is quite comparable to t-MN incidence after chemotherapy or auto-HSCT for NHL patients. In our centers, the incidence of t-MN after CAR T therapy was 3.2%. According to literature review, incidences of t-MN following CAR T therapy varied from less than 1% to 12.9%. In comparison, up to 10% of NHL patients may develop t-MN within 10 years after chemotherapy or auto-HSCT (41), though with a pretty longer follow-up. Thus, according to current data of incidence, it seems likely to infer that CAR T cell infusion doesn’t significantly increase the risk of t-MN. However, multiple factors could contribute to the evident discrepancy in t-MN incidence after CAR T therapy, for example, the differences in prior cytotoxic therapies, duration, accuracy and completeness of follow-up, and even different CAR T products. It is reasonable that the possibility of developing t-MN will increase with longer follow-up. For example, for patients receiving auto-HSCT, t-MN incidence varied from 1% in 30 months (42) to 11.7% in 6 years (43). Besides, as elaborated in table 1 and table 3, various CAR T products were included, which suggested the development of t-MN was an unbiased and potential risk of these genetically modified cellular products, albeit the risks for different cell products are still unclear.

Accordingly, an important question has been the extent to which the risk of t-MN relates to previous cytotoxic therapies with leukemogenic potential, or the extent to which the risk results directly from CAR T therapy per se. Not surprisingly, BMA prior to CAR T therapy would provide new insights into the underlying state of hematopoiesis at baseline. However, as a matter of fact, BMA including genetic testing is not regularly implemented in clinic for lymphoma patients without BM involvement or cytopenia, so it is difficult to clarify the existence of CHIP before cell infusion. For all of our new cases, only one patient received BMA before cell infusion due to cytopenia, nevertheless, genetic testing was scarce. Though Cordeiro et al. (20) and Strati et al. (23) reported that 2 t-MDS patients (n=8) had ascertained the pre-MDS state before CAR T therapy, regrettably, the majority of studies lacked the results of BMA at baseline. Besides, it is difficult to prove whether pretreatment alone enough to drive disease progression from CHIP to t-MN.

On the other hand, it still remains unclear whether CAR T cell itself contributes to t-MN, and if so by which mechanism. It is interesting to note a case report that a patient with B-cell acute lymphoblastic leukemia (B-ALL) relapsed 9 months after CAR T cell infusion, which was caused by unintentional introduction of CAR gene into a single leukemic B cell during T cell manufacturing (44). Insertional oncogenesis due to insertion of a viral vector near an oncogene in the engineered T-cell is a possibility of second cancers. However, as far as we know, no late event of T cell malignancy following CAR T therapy has ever been reported up to date.

It has been well recognized that myeloid neoplasm is a heterogeneous disease that arises from genetic alterations in HSC which resides within the complex and dysfunctional BM microenvironment (45, 46). To characterize the cell type composition and status in BM of t-MN patients after CAR T therapy, BM samples from a t-MDS patient and a HD were assessed. First, we identified 14 cell populations in t-MDS BM, as same as the normal BM (**Figure 2B**). There were no distinct clusters detected, suggesting much of the normal transcriptional hierarchy was still reflected in the t-MDS patient. Moreover, our scRNA-seq data demonstrated differential gene expression and biologic pathways in HSC/CMP populations between t-MDS and HD (**Figure 3** and **Table 2**), among which several genes have previously been reported in MDS patients. For example, IFI27 involves in the IRF4 regulatory network, which is implicated in MDS, leukemia, myeloma and lymphoma (12, 47). PRDX2, a member of the peroxiredoxin family, is markedly upregulated and involves in the pathogenesis of refractory cytopenia with multilineage dysplasia (16). Thus, there are multiple transcriptional similarities between p-MDS and t-MDS after CAR T therapy. Furthermore, given that the dysfunctional immune system plays a prominent role in BM microenvironment and is necessary for modulating tumorigenesis and progression, we also characterized T/NK subpopulations of t-MDS BM and identified 13 clusters (**Figure 4B**). The t-MDS aspirate tended to contain much more T cells and CTL and had a higher CTL/T ratio (**Figures 4D, E**), which was consistent with prior reports concerning p-MDS, although evidence showed that this activated response facilitated the killing of normal HSPC and acceletated MDS development (46, 48). In summary, based on these scRNA-seq data and published literature, there are several similarities between t-MDS and p-MDS, including transcriptional changes of the HSPC/CMP population and increased CTL ratio in BM. However, due to limited cell numbers from the sole t-MDS BM sample, the unique cell types and transcriptional signatures of t-MDS after CAR T therapy remain unclear. In the future, further analysis of scRNA-seq data from more t-MDS and p-MDS BM samples hold the promise to elucidate how different cell types contribute to t-MDS progression after CAR T therapy, and provide directions to recognizing t-MN regulators and biomarkers for precision medicine and prognosis evaluation.

Apart from t-MN, other sorts of second cancers were also reported. The real-world study of axi-cel within 1297 LBCL patients elaborated the entire incidence of second cancers was 4% (n=50), among which non-myeloid neoplasms accounted for 62% and squamous cell skin malignancy accounted for 22% (24). Another study of CD19 CAR T within 86 patients reported a pretty higher incidence of second cancers was 15% (n=13), among which non-myeloid neoplasms accounted for 69% and non-melanoma skin cancer accounted for 46% (20). In both studies, non-melanoma skin cancer emerged as the most common non-myeloid neoplasms. Besides, multiple myeloma, melanoma and non-invasive bladder cancer were also reported. Multiple occurrences of second cancers were also reported in some patients.

To date, there is no reports focused on t-MN complications after CAR T therapy. In the near future, long-term follow-up of ongoing clinical trials and commercial CAR T products will be needed to estimate the incidence of t-MN. As a matter of fact, FDA has mandated more than 15-year follow-up for axi-cel and tisa-gen, and prospective post-marketing registry studies have been proceeding by CIBMTR with a key endpoint designed as the incidence of second cancer. Most importantly, in order to determine the extent to which prior cytotoxic therapies vs. CAR T therapy contributes to t-MN, it is important to launch a cohort study comparing the risk of t-MN among CAR T vs. non-CAR T R/R B-cell lymphoma populations.

Taken together, long-term and close monitoring for SMN in patients receiving CAR T therapy is necessary. Moreover, researches are needed for a better comprehension of pathophysiology of SMN following CAR T therapy in the future to establish evidence-based risk stratification and treatment approach.

## Data availability statement

The original contributions presented in the study are included in the article/**Supplementary Material**. Further inquiries can be directed to the corresponding authors.

## Ethics statement

The studies involving human participants were reviewed and approved by the Ethics Committee of the Second Affiliated Hospital, Zhejiang University (Hangzhou, China). The patients/participants provided their written informed consent to participate in this study. Written informed consent was obtained from the individual(s) for the publication of any potentially identifiable images or data included in this article.

## Author contributions

Conceptualization, PL and HL. Data collection: MZ. Data analysis and interpretation: AZ. Writing-Original Draft Preparation, AZ and MZ. Writing-review and editing: WQ and AL. All authors contributed to the article and approved the submitted version.
